# Prevalence of Falls in Patients Presenting to an Ophthalmic Outpatients Department- A Surveillance Study

**DOI:** 10.22599/bioj.178

**Published:** 2021-08-20

**Authors:** Jignasa Mehta, Karen Knowles, Erin Wilson

**Affiliations:** 1 University of Liverpool, GB; 2 Southport and Ormskirk Hospital NHS Trust, GB; 3 Wirral University Teaching Hospital NHS Foundation Trust, GB

**Keywords:** Falls, Vision, Ophthalmic conditions

## Abstract

**Introduction:**

Approximately 1 in 3 adults aged 65 and over experience a fall each year. Poor vision is an identified risk factor. The aim of this cross-sectional public health surveillance audit was to determine the prevalence of falls experienced in the previous 12 months by adult patients presenting to an ophthalmology department.

**Methods:**

A short questionnaire was given to consecutive patients attending an ophthalmology department at two trusts in the North West to determine whether they had experienced a fall in the previous 12 months, whether they had suffered a fracture, their eye condition and the type of glasses worn.

**Results:**

Data was collected for 585 patients (mean age: 69 years, SD: 13.8). Falls in the previous 12 months were reported by 96 (16.4%) patients, and a significant proportion of these were aged 60 years and over (82%, p < 0.0001, one-sample binomial test). Half of the respondents were unaware of their eye health condition for which they were attending the department. Glaucoma was the most prevalent condition in those who had experienced a fall (43%). A significant proportion of the individuals who reported a fall wore single-vision glasses (43%, p < 0.0001, one-sample chi-square test).

**Conclusion:**

In an ophthalmology outpatient department, the proportion of older adults who experienced a fall in the previous 12 months was lower than the reported prevalence in the general population. There is a need for health literacy to educate patients about their eye condition, the potential effects on their visual function and, consequently, managing falls risk.

## Introduction

Falls are experienced every year in approximately 20–33% of community-dwelling older adults (Peel 2011). The population in the UK is ageing; currently, there are 12 million people in the UK aged 65 and above, and by 2066 this could increase to over 20 million (ONS 2018). With this increase in demographic ageing, the prevalence of sight loss and blindness may increase from 3.0% to 5.4% by 2050 (Pezzullo et al. 2018). Furthermore, as the evidence shows that the risk of falls increases with age (Klein et al. 2003), we need to address the association of both vision and falls as a matter of public health concern. The annual hospital cost for all incident hip fractures in individuals aged 60 years in the UK is estimated to be £1.1 billion (Leal et al. 2016) and does not include onward social care. A briefing paper published by the RNIB estimated that the cost of falls associated with sight loss to the NHS is £25.1 million per year (Boyce 2011). Along with the financial implications of falls to the NHS, the consequences to the individual’s quality of life are more far-reaching, including loss of independence, increased depression and reduced mobility (Salkeld et al. 2000).

Physiologically, to maintain balance and stay upright we need appropriate inputs from the visual, proprioceptive and somatosensory systems; therefore, any deficient input from one of these systems could potentially lead to postural instability and subsequently a fall (Black & Wood 2005). Therefore, the impact of impaired vision in falls is crucial, and research has shown the association between reduced visual functions and falls in older adults (de Boer et al. 2004; Freeman et al. 2007; Ivers et al. 2003; Klein et al. 2003; Lord, Clark & Webster 1991; Lord & Dayhew 2001; Nevitt et al. 1989; Tinetti, Speechley & Ginter 1988). Several studies have also examined the association between age-related ophthalmological conditions and falls.

Increased risk of falls has been reported in individuals with age-related macular degeneration (Chung et al. 2017; Radvay et al. 2007; Szabo et al. 2010), glaucoma (Black, Wood & Lovie-Kitchin 2011; Haymes et al. 2007; Tanabe et al. 2012) and cataracts (Ivers et al. 1998; Palagyi et al. 2016). Studies have reported a reduction in falls risk following first eye surgery (Harwood et al. 2005; Palagyi et al. 2017a; To et al. 2014). In contrast, an increase in falls has been reported between first and second eye surgery (Meuleners et al. 2014), which may cause a deficit in stereovision. A further modifiable visual risk factor that has been linked to an increased risk of falls, and in particular trips, is the use of varifocal glasses (Davies et al. 2001; Lord, Dayhew & Howland 2002) when compared to wearing distance single-vision spectacles (Johnson et al. 2007). Haran et al. (2010) also found that older adults who took part in regular outdoor activity experienced fewer falls when wearing single-vision glasses.

Whilst we have highlighted several cohort studies that have identified specific visual risk factors for falls and falls risk in older adults with ophthalmological conditions, there is no study to identify the prevalence of falls in patients presenting to an ophthalmology outpatient department. We also understand that a history of falling is a strong risk factor for a further fall (Deandrea et al. 2010; Gale et al. 2018; Pohl et al. 2014). Therefore, prior to designing a case-control study to measure clinical visual functions to determine the risk of falls, we conducted a surveillance audit to determine the prevalence of falls in patients attending an ophthalmology outpatient department. In addition, the audit would highlight the need for ophthalmic health professionals to identify patients at risk of further falls as per the new quality statement in the NICE guidance (NICE 2017): ‘’Older people are asked about falls when they have routine assessments and reviews with health and social care practitioners, and if they present at a hospital’.

## Methods

A public health surveillance initiative to identify the prevalence of falls in adult patients (>18 years) attending the ophthalmology unit led to the development of a short questionnaire in collaboration with the falls multi-disciplinary team at Southport and Ormskirk NHS Trust. The questionnaire was purposefully kept brief to not make it too onerous for the patient. The aim was for patients to self-report any falls that they had in the previous 12 months, fractures that may have occurred as a result of the fall, the type of glasses they wore and their eye condition.

The questionnaire was completed at two NHS hospital trusts: Southport and Ormskirk Hospital Trust and Warrington and Halton Hospitals Trust. The ophthalmology reception staff for each trust administered the forms to patients attending the ophthalmology outpatients. The data was collected for one month over two time points for each site. This data was then anonymised, collated across both sites and analysed using descriptive statistics, ANOVA and chi-square analysis. Local trust approval was obtained at both Southport and Ormskirk Hospital Trust and Warrington and Halton Hospitals Trust. Ethical approval and informed consent were not required for this audit.

## Results

**Figure 1:**
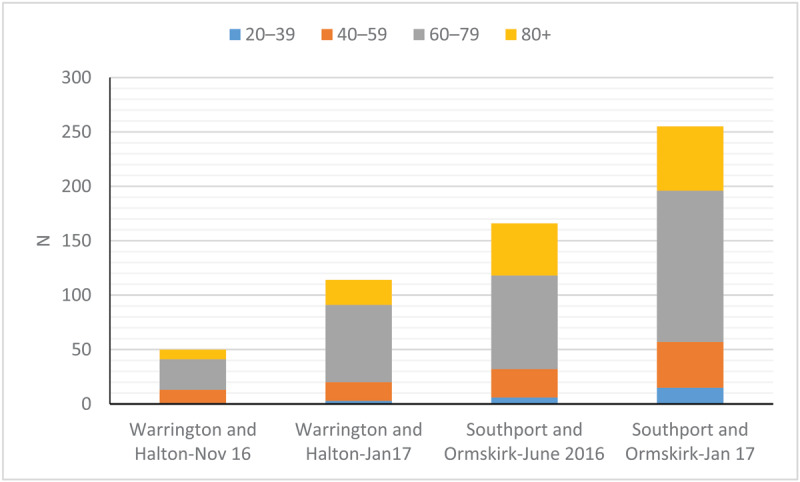
Age distribution of respondents from each site.

A total of 585 patients with a mean age of 69 years (range 20–102 years, SD 13.8) reported their falls history from the previous 12 months from both trusts. There was no significant difference in the age of the respondents across the sites and time periods they were surveyed (***Figure 1***, p > 0.05, one-way ANOVA). A significant proportion of responders were older adults (aged 60 years and over) (N = 463 (79%) vs N = 122 (21%), p < 0.0002, one-sample binomial test).

**Figure 2:**
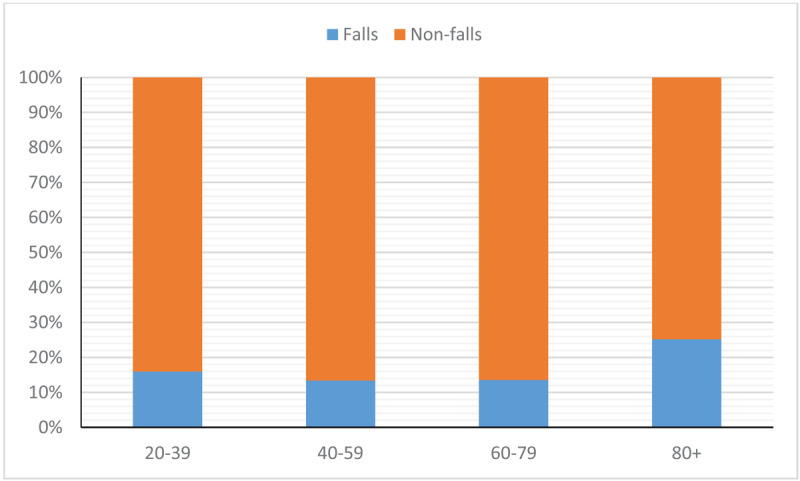
Proportion of respondents who did and did not report a fall in the previous 12 months across the different age groups.

A total of 96 respondents (16.4%) had experienced at least one fall in the previous year. ***Figure 2*** illustrates the proportion of patients within each age group of the entire sample who experienced a fall. A significant proportion of the falls respondents were older adults (N = 79, 82%, p < 0.0001, one-sample binomial test), and of these, 44% (N = 35) were aged 80 years and over. Thirty-nine patients (41%) had experienced multiple (two or more) falls, and a significant proportion of these individuals were seen in Southport and Ormskirk (69%) compared to Warrington and Halton (31%) (p = 0.0009, chi-square). Twelve respondents experienced a fracture (mean age: 72 years, SD: 13.5, range 39–84).

Patients were asked to self-report their eye condition, and over half of the respondents (N = 297, 50.7%) did not record this information. Glaucoma was the most commonly self-reported condition (N = 116), which was reported either as an isolated condition or with cataracts, AMD or Sjorgren’s syndrome. This was followed by cataracts (N = 55), which also featured alongside other ophthalmological conditions. Data was cross-referenced against the patient records to determine the presence of key age-related ophthalmic conditions: cataracts, AMD and glaucoma (***Table 1***). No data were obtained for six respondents, and the remaining had other conditions (N = 160).

**Table 1:** Prevalence of key ophthalmic conditions in the respondents recorded from the hospital records.


Age-related ophthalmic condition	N (%)

Cataracts	149 (25.5)

AMD	36 (6.2)

Glaucoma	234 (40.0)

Other	160 (27.3)

Missing data	6 (1)


Two thirds of respondents aged 60 and over presented with at least one key age-related ophthalmic condition (N = 306, 66%), and the remaining one third had other conditions or were under review for suspect age-related ophthalmic conditions. Amongst the older adults who had experienced a fall, 68% (N = 54) had at least one of the key ophthalmic conditions compared to 32% (N = 25) who had other conditions (p = 0.002, one-sample binomial test).

**Figure 3:**
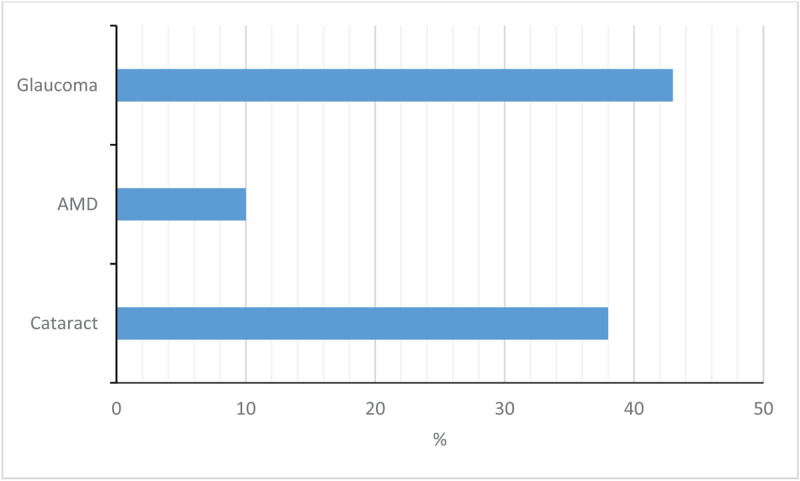
Percentage of respondents (aged 60 and over) with key age-related ophthalmic conditions who had experienced a fall in the previous 12 months.

Many of the older adults had co-existing age-related ophthalmic conditions. The most prevalent condition was glaucoma, followed by cataracts in the older adults who had experienced a fall in the previous 12 months (***Figure 3***).

Varifocals (30.6%) and single-vision glasses (35.7%) were commonly worn by all respondents (***Table 2***). On further analysis of the sample who had experienced a fall, a significant proportion wore single-vision glasses compared to bifocals, varifocals and no glasses (p < 0.0001, one-sample chi-square test, ***Table 2***).

**Table 2:** Type of glasses worn by respondents (N = 585) and those reporting a fall in the previous 12 months (N = 96).


TYPES OF GLASSES WORN	N (%) ALL RESPONDENTS	N (%) RESPONDENTS REPORTING A FALL

No glasses	99 (16.9)	14 (15.1)

Single-vision glasses	209 (35.7)	40 (43.0)

Varifocals	179 (30.6)	23 (24.7)

Bifocals	77 (13.2)	16 (17.2)

Not reported	21 (3.6)	3 (3)


## Discussion

The results of our cross-sectional surveillance audit demonstrate that 17% of all the older adult patients (60 years and over) attending an ophthalmology outpatient department at two North West Trusts reported having had a fall in the previous 12 months. The proportion of falls in the older adult sample is contrary to the commonly reported statistic of one third of older adults aged 65 and over experiencing at least one fall per year and rising to 50% of people older than 80 years (NICE 2013). This low prevalence seen in our surveillance audit may be explained by the reluctance of older adults to ‘go public’ about their fall due to fear of added pressure to change their lifestyle and threat to their independence (Kingston 2000; Yardley et al. 2006). Also, the under-reporting of falls may be due to the cross-section nature of our data and lack of cognitive function measure contributing to recall bias (Freiberger & de Vreede 2011).

A further limitation when capturing the data was that the fall was not defined. Therefore, participants may not have recognized and reported a fall that was a slip, trip, near miss or non-injurious (Freiberger & de Vreede 2011). Hence, in future studies to improve the reporting of falls, a diary or calendar should be used to collect prospective data using the Prevention of Falls Network Europe (ProFaNE) definition of a fall as ‘an unexpected event in which the participant comes to rest on the ground, floor, or lower-level’ (Lamb et al. 2005).

Furthermore, in a survey-based study of 33,104 adults over 18 years of age, self-reported moderate rather than severe visual impairment was reported to be associated with injurious falls (OR 1.58, 95%CI: 1.15–2.17) (French et al. 2016). There were fewer individuals with severe visual impairment who experienced an injurious fall possibly due to them adopting a more cautious approach to ambulation. This was confirmed in a qualitative study exploring the fear of falling in older adults with age-related ophthalmic conditions where the participants reported taking more care and being cautious (Mehta 2020) and could further account for the fewer number of falls reported in this study. Future studies could examine the prevalence of falls in older adults in other outpatient departments and conduct case-control studies to establish whether individuals with objectively assessed mild, moderate and severe visual impairment and/or deficit visual functions are at greater risk of falls.

The respondents’ lack of awareness of their ophthalmic condition was evident in over half of the patients. This finding points to the need to improve the ability of individuals to access and understand their condition as low levels of health literacy have been reported to be associated with poor health outcomes and increased healthcare costs (Easton, Entwistle & Williams 2010). A qualitative piece of work exploring the fear of falling in older adults with age-related ophthalmic conditions reported the importance of knowledge as cultural capital in managing risk or fear of falling (Mehta 2020). Hence, further work should aim to evaluate the effect of health literacy and information on managing the risk of falls. Falls risk in older adults can also be explored in those who are aware of their condition to determine if they take extra precautions by altering gait and gaze when navigating their environment. Falls and fear of falling have been reported to be predictors of each other and have common shared risk factors, for example being female, history of stroke, visual impairment and a sedentary lifestyle (Friedman et al. 2002; Murphy, Dubin & Gill 2003). A few studies have reported the association of increased fear of falling with specific ophthalmic conditions, for example, glaucoma, AMD, cataracts and diabetic retinopathy (Adachi et al. 2018; Hewston & Deshpande 2018; Palagyi et al. 2017b).

The prevalence of age-related ophthalmic conditions, namely cataracts, glaucoma and AMD, increases with age. Older adults aged 80 years have one third of all cases of cataract, glaucoma and AMD (Klein & Klein 2013). In our study, glaucoma was the most common presenting condition in older adults (aged 60 years and over) followed by cataracts. Both glaucoma and cataracts can potentially be associated with reduced visual fields and stereopsis, respectively. In our study, of those who had a fall, twice as many of the patients aged 60 and over had at least one of the key age-related ophthalmic conditions, yet there was no significant association between a fall in the previous 12 months and the presence of an age-related ophthalmic condition. A limitation of this study is the lack of clinical data on measured visual function to determine the association between falls and impaired visual function.

Unlike previously reported evidence on the association between varifocals and the increased risk of falls (Davies et al. 2001; Lord, Dayhew & Howland 2002), our data did not demonstrate an association between the use of varifocals and falls, and instead, a greater proportion of those who had fallen wore single-vision glasses. It is conceivable that respondents did not always wear their single-vision glasses at all times, and this postulation would require further study.

## Conclusion

The prevalence of falls in older adult patients attending an ophthalmology outpatient department in our audit was less than that reported in the general older adult population. Impaired vision is a recognised risk factor for falls, but this is potentially the case in those who have undetected visual defects and are not under any ophthalmic care. Future work could explore whether ophthalmic patients adopt a more cautious modified approach to ambulation due to their vision and therefore experience fewer falls. However, falls remain to be an issue in older age groups. Irrespective of the prevalence, ophthalmic health professionals are in an ideal position for falls case/risk identification and onward referral specialist assessment to prevent further falls. Furthermore, this surveillance study highlights that people have poor health awareness. As healthcare professionals, we need to consider health literacy in communicating to patients their ophthalmic diagnosis and potential effects on their vision to increase their understanding of their condition.
